# Mechanisms of Modulation of Mitochondrial Architecture

**DOI:** 10.3390/biom13081225

**Published:** 2023-08-07

**Authors:** Juan Pablo Muñoz, Fernanda Luisa Basei, María Laura Rojas, David Galvis, Antonio Zorzano

**Affiliations:** 1 CIBER de Diabetes y Enfermedades Metabólicas Asociadas (CIBERDEM), 28029 Madrid, Spain; 2 Institut d’Investigació Biomèdica Sant Pau (IIB SANT PAU), 08041 Barcelona, Spain; 3 Faculdade de Ciências Farmacêuticas, Universidade Estadual de Campinas, 13083-871 Campinas, SP, Brazil; 4 Centro de Investigaciones en Bioquímica Clínica e Inmunología (CIBICI), Facultad de Ciencias Químicas, Universidad Nacional de Córdoba, Córdoba X5000HUA, Argentina; 5 Programa de Química Farmacéutica, Universidad CES, Medellín 050031, Colombia; 6 Institute for Research in Biomedicine (IRB Barcelona), 08028 Barcelona, Spain; 7 Departament de Bioquímica i Biomedicina Molecular, Facultat de Biologia, Universitat de Barcelona, 08028 Barcelona, Spain

**Keywords:** mitochondria, metabolism, mitochondrial dynamics, pharmacology, lipids, metabolic disease, membrane contact sites (MCSs), tethers, post-translational modification

## Abstract

Mitochondrial network architecture plays a critical role in cellular physiology. Indeed, alterations in the shape of mitochondria upon exposure to cellular stress can cause the dysfunction of these organelles. In this scenario, mitochondrial dynamics proteins and the phospholipid composition of the mitochondrial membrane are key for fine-tuning the modulation of mitochondrial architecture. In addition, several factors including post-translational modifications such as the phosphorylation, acetylation, SUMOylation, and o-GlcNAcylation of mitochondrial dynamics proteins contribute to shaping the plasticity of this architecture. In this regard, several studies have evidenced that, upon metabolic stress, mitochondrial dynamics proteins are post-translationally modified, leading to the alteration of mitochondrial architecture. Interestingly, several proteins that sustain the mitochondrial lipid composition also modulate mitochondrial morphology and organelle communication. In this context, pharmacological studies have revealed that the modulation of mitochondrial shape and function emerges as a potential therapeutic strategy for metabolic diseases. Here, we review the factors that modulate mitochondrial architecture.

## 1. Mitochondrial Network Architecture

Mitochondrial architecture is determined by several components, which include the following: mitochondrial distribution in the cytosol, supported by interaction with the cytoskeleton; events of fission and fusion, mediated by mitochondrial dynamics proteins; mitochondrial network contact with other organelles (e.g., endoplasmic reticulum (ER), lipid droplets (LDs), lysosomes, and plasma membrane); and the lipid composition of mitochondrial membranes [1,2,3,4,5,6,7,8].

In addition, the ultrastructure of mitochondria may be modified by mitochondrial ion transport fluxes such as Ca^2+^ and K^+^ [9,10]. An influx of these ions can alter the morphology of these organelles, leading to swelling or a donut-like shape [4,11].

*Cytoskeleton:* Recent work evidenced that cytoskeleton elements differentially modulate the mobility and shape of mitochondria [7,12]. Nocodazole, a drug that disrupts microtubule polymerization, reduces mitochondrial network cellular coverage, disrupts mitochondrial alignment with microtubules, and decreases mitochondrial mobility. In contrast, F-actin or intermediate filaments maintain mitochondria confined to the microtubule network, and their disruption alters mitochondrial shape [7,12]. Of note, it has been reported that ARP2/3 and Formin-dependent actin cycling (actin assembly and disassembly) occur through the mitochondrial network [1]. Furthermore, actin polymerization promotes mitochondrial fission, whereas F-actin disruption causes mitochondria fusion [1]. In this regard, F-actin is assembled into the outer mitochondrial membrane (OMM), thereby contributing to mitochondrial fission [1,13].

*Mitochondrial dynamics:* Continual shifting of mitochondrial network architecture is supported by mitochondrial dynamics proteins. In the last 20 years, several proteins that coordinate mitochondrial fission and fusion events have been defined [14,15,16,17,18]. Moreover, recent studies have indicated that mitochondrial fission and fusion occur in the same site on the mitochondria [2], and also that ER wrapping around mitochondria is required to sustain the dynamics of these organelles [6]. A recent report describes two distinct types of mitochondrial fission. Peripheral fission sustains mitochondrial degradation mediated by mitophagy while midzone mitochondrial fission is required to maintain mitochondrial biogenesis and dynamics [19] (see Section 2).

*Mitochondrial contacts with other organelles.* A growing number of studies have demonstrated the interaction of mitochondria with different organelles. This is an emergent field of research that will permit understanding of how some metabolic and cell signaling mechanisms originate.

*Mitochondria–endoplasmic reticulum contacts.* Several investigations have reported that mitochondria–endoplasmic reticulum interactions are required to modulate the calcium signal. Close contact between mitochondria and endoplasmic reticulum plays a critical role in mitochondrial calcium uptake. For instance, the complex VDAC1-GRP75-PI3R mediates mitochondria–endoplasmic reticulum tethering and is required to sustain mitochondrial calcium homeostasis [20]. Moreover, mitochondria–endoplasmic reticulum interaction is involved in apoptosis and autophagy activation [21,22,23]. Overnutrition induced via a high fat diet in mice leads to alteration in calcium handling in the liver [24,25]. These studies have demonstrated that mitochondrial–endoplasmic reticulum contacts are disrupted upon ingestion of a high fat diet. On the other hand, the mechanism of phosphatidylethanolamine (PE) synthesis and shuttling requires mitochondrial–endoplasmic reticulum contacts. The enzymes required for phosphatidic acid (PA) conversion to PE are localized in mitochondrial–endoplasmic reticulum contact. Several proteins contribute to mitochondria–endoplasmic reticulum tethering and the maintenance of calcium transfer and lipid synthesis (revised by Wenzel et al., 2022) [26].

*Mitochondria–lipid droplets contacts.* Contacts between mitochondria and lipid droplets (LDs) are mediated by several proteins including PLIN1, PLIN5, MFN2, and MIGA2 [27] (see Section 4). The highly dynamic interactions between both organelles allow fatty acid migration from LD to mitochondria, where it is oxidized to produce ATP or heat. Furthermore, they are also needed for LD expansion, which stores fatty acids and lipid intermediates to avoid cellular lipotoxicity [28,29].

*Mitochondria–peroxisome contacts.* Mitochondrial–peroxisome interaction plays a relevant role in fatty acid metabolism and in cellular redox homeostasis; a coordinate function of these organelles is required to sustain mitochondrial activity. Studies have shown that peroxisomal protein (PEX5 or PEX16) ablation in mice livers led to mitochondrial dysfunction and fragmentation [30,31]. Moreover, PEX16 knockout mice showed that peroxisomes are required to sustain mitochondrial homeostasis upon metabolic stress induced by a high fat diet [31]. Two proteins involved in mitochondria–peroxisome tethering have been identified, enoyl-CoA-δ isomerase 2 (ECI2) and the lipid transport protein VPS13D [32,33]. Interestingly, knockdown of VPS13D expression promotes mitochondrial fragmentation, revealing the role of this protein in mitochondrial architecture [34]. Also interesting, a recent study of yeast demonstrated that PEX4 and Fzo1 (the yeast orthologue of MFN1 and MFN2 proteins) are involved in mitochondria–peroxisome tethering [35]. Further investigations are required to elucidate the role of this protein in mammalian cells.

*Mitochondria–endosome contacts.* A recent report has documented that VDAC2 tethers RAS-PI3K-positive early endosomes with mitochondria [36]. This study also shows that this interaction promotes endosome acidification and maturation [36]. Depletion of the mitochondrial fusion protein MFN1 promotes the association of endosomes and mitochondria through a process that requires Rab5C [37]. In addition, it has been described that endosomes are in close contact with mitochondria in axons of retinal ganglion cells [38]. Rab7a is localized in endosomes, and ribonucleoprotein particles and mitochondria are in contact in axons. In this way, these contacts are required for local translation of mRNA that encodes to mitochondrial proteins. Moreover, Rab7a mutations lead to alteration of mitochondrial morphology, decrease mitochondrial membrane potential, and modify the mitochondrial retrograde and anterograde transport in axons [38].

*Mitochondria–lysosome contacts.* Lysosome–mitochondrial tethering is mediated by Rab7, is localized in lysosomes, and is modulated by TBC1D15, a Rab7 GTPase activating protein, and Fis1, localized in mitochondria [39]. This study also documented that lysosomes localize in mitochondrial fission sites, suggesting that they modulate mitochondrial dynamics [39]. The functional role of mitochondrial–lysosome tethering on calcium homeostasis has recently been reported [40]. The cation channel TRPML1 localized in lysosomes and late endosomes mediates the direct calcium transfer into mitochondria. Moreover, TRPML1 loss-of-function impairs calcium transfer, and it results in alterations of tethering dynamics [40].

Mitochondrial architecture is altered by environmental stimuli. It has been widely described in cell models that metabolic stress, induced by OXPHOS inhibitors, nutrient starvation, endoplasmic reticulum (ER) stressors, protein and RNA synthesis suppressors, and UV irradiation, induce transient mitochondrial elongation upon acute treatment [41,42,43,44]. Moreover, mitochondrial elongation improves the OXPHOS activity under these stimuli [41,45]. It has been demonstrated that the relationship between mitochondrial morphology and OXPHOS is complex. Given that, maximal mitochondrial respiration is carried out upon mitochondrial uncoupling agents (such as CCCP) treatment and these compounds promote mitochondrial depolarization and fragmentation. A simple relation between mitochondrial elongation and increased OXPHOS activity may not occur under all conditions. Thus, genetic ablation of the mitochondrial fusion protein Mitofusin 1, in which the mitochondrial network is fragmented, showed similar OXPHOS activity in glucose- or galactose-supplemented culture media [46]. In keeping with this, it has been reported that hepatic MFN1 ablation increases mitochondrial mass and OXPHOS activity [47]. Recent evidence has shed new light on the link between the morphology and function of mitochondria. Ngo et al. demonstrated that mitochondrial fragmentation enhances long-chain fatty acid oxidation. Moreover, mitochondrial fragmentation decreases the inhibition of malonyl-CoA-dependent carnitine palmitoyltransferase I. These data reveal that mitochondrial fragmentation upon lipid overload activates mitochondrial β-oxidation [48].

Mitochondrial shape alterations have been reported in conditions such as obesity, diabetes, ischemic–reperfusion, senescence, and cancer [24,49,50,51,52,53,54]. Under metabolic stress induced by nutrient overload, mitochondrial network architecture is fragmented, and this alteration is associated with a metabolic dysfunction that results in a decrease in OXPHOS activity, apoptosis, or mitophagy [55]. These alterations have been evidenced in several cell models, including β-pancreatic cells, skeletal muscle cells, cardiomyocytes, and adipocytes [56,57,58,59,60]. Similarly, disruption of the mitochondrial network architecture and mitochondrial dysfunction have been reported based on in vivo mouse models of diabetes, cardiomyopathy, non-alcoholic fatty liver disease, and obesity [51,61,62,63]. Emergent evidence indicates that mitophagy is impaired in metabolic diseases. The perturbation of mitochondrial homeostasis that causes a decrease in mitochondrial membrane potential induces mitochondrial fragmentation and activates lysosome-dependent mitochondrial degradation [55,64,65]. Recently, Han and colleagues demonstrated the presence of distinct populations of mitochondria in non-small cell lung cancer tumors. They reported a peri-droplet mitochondrial network throughout the cytoplasm in oxidative lung adenocarcinoma (LUAD) cells that was accompanied by an increase in fatty acid oxidation. On the other hand, cells with higher glycolytic activity (lung squamous cell carcinoma) show perinuclear mitochondria. These exciting results support the importance of mitochondrial architecture in sustaining tumor metabolism [54]. Thus, modulation of mitochondrial architecture appears to play a crucial role in cellular responses leading to metabolic diseases.

The discovery of proteins that modulate mitochondrial architecture and the mechanism of action of the same has allowed intense research efforts regarding the link between metabolism and mitochondrial function. These efforts have revealed that changes in the expression or post-translational modification of mitochondrial dynamics proteins are associated with metabolic diseases. As a result of these investigations, new fields of drug discovery aiming to modulate mitochondrial dynamics and thereby redirect cellular metabolism have emerged.

Given the continuous and rapid changes in mitochondrial architecture, sophisticated microscopy approaches are required to elucidate the biological factors that regulate mitochondrial morphology in vivo. Recent studies using super-resolution microscopy have revealed the cellular mechanisms by which mitochondrial network architecture is modulated [2,5,6,19]. In this regard, live-cell imaging has shown that ER–mitochondrial constriction sites prime and coordinate the mitochondrial fission and fusion protein machinery [2]. Remarkably, recent evidence using proximal proteomic and live-cell microscopy demonstrates that ABHD16A, a hydrolase enzyme that mediates the conversion of phosphatidylserine (PS) to lysophosphatidylserine (lysoPS), is localized at ER–mitochondria contacts and is required for mitochondrial fission and fusion [8].

**Figure 1:**
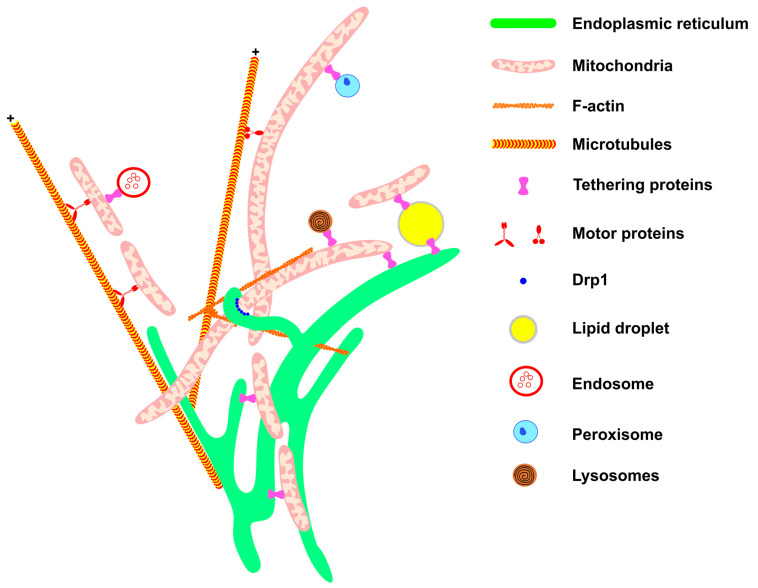
Mitochondrial network architecture. Mitochondria morphology is sustained and affected by interactions with other cellular structures. Communication with the ER (green) provides mitochondrial phospholipids and sustains calcium homeostasis. Interaction with the components of the cytoskeleton (F-actin filaments and microtubules, in orange) moves the mitochondria to specific regions of the cell, such as the appropriate location for cell division, or the periphery of the cell in the case of neurons. Motor protein kinesins and dyneins help to move mitochondria along the cytoskeletons of microtubules (for review, see McElroy et al., 2023) [70]. The endoplasmic reticulum wraps the mitochondria, thereby helping to recruit DRP1 (blue) on the mitochondrial membrane. Through communication with LDs (yellow), mitochondria are provided with fatty acids for β-oxidation and in turn provide energy for LD expansion. The contact between mitochondria and endosome (red) is important for mitochondria quality control through lipid and ion transference. Peroxisome (blue) and mitochondria communication allows the complementation of fatty acid oxidation and anti-oxidant system activation (for review, see Chen et al., 2020) [71]. Mitochondria–lysosome interaction (red and black) facilitates the direct transference of calcium from lysosomes to mitochondria. All these interactions are mediated by specific proteins that connect or tether the membranes.

The use of intravital multiphoton microscopy-based technology, which allows collection of cell images in live animals, has revealed that the mitochondrial network is disrupted upon pathological conditions in vivo. This work has demonstrated the transient disruption of the mitochondrial network upon brain ischemia. The mitochondria of dendrites at pyramidal neurons have an elongated shape, as revealed by intravital microscopy and electron microscopy. In contrast, upon transient brain ischemia, mitochondria undergo fragmentation and have a globular morphology, and “mitochondria-on-a-string” (MOAS) structures appear. Ischemic recovery rescues mitochondrial elongation and decreases globular mitochondria, and MOAS morphology is not observed in dendrites [66]. Taken together, these observations indicate that a transient and reversible alteration of mitochondrial morphology occurs under ischemia in vivo. Interestingly, MOAS morphology has also been described in neurons in cellular and mouse models of Alzheimer’s disease, aging, and hypoxia. It has been proposed that energetic collapse upon ischemic injury leads to unfinished mitochondrial fission [67,68]. Intravital microscopy has also been used to study live-cell mitochondrial dynamics and mitophagy in the livers of mice after acute ethanol treatment [68]. This report demonstrated that this treatment disrupts mitochondrial membrane potential; however, only a subpopulation of mitochondria undergoes mitophagy. In addition, ethanol activates selective mitophagy instead of lipophagy in the livers of this model [69]. In this context, a novel state-of-the-art methodological approach will unravel the function and modification of the mitochondrial network under physiological and pathological conditions (Figure 1 and Table 1).

## 2. Mitochondrial Dynamics Proteins

The function of mitochondrial dynamics proteins is modulated by protein expression, proteolytic processing, subcellular localization, and post-translational modifications. Additionally, some accessory mitochondrial proteins and mitochondrial lipid composition help to modulate mitochondrial shape. Thus, the fine-tuned coordination of these factors regulates the architecture and function of the mitochondrial network. Mitochondrial dynamics proteins have been widely studied, and their function has been characterized in several tissues under a range of physiological and pathological conditions. In this section, we will describe the major and recent advances in the field of mitochondrial dynamics.

### 2.1. Mitochondrial Fission Proteins

Mitochondrial fission is characterized by the division of a mitochondrion into two smaller mitochondria. This process is key for mitochondrial distribution during mitosis, mitochondrial clearance, ATP distribution (e.g., distal axons), and metabolism [48,64,77,78]. The main determinant protein for fission is the dynamin-related protein 1 (DRP1), which, together with adaptor proteins (FIS1 and MFF), the ER, and the cytoskeleton, promotes mitochondrial constriction and fragmentation [6]. It has been demonstrated that β-cells and brown adipocytes present predominantly mitochondrial fission under physiological stimulus or nutrient exposition; moreover, mitochondrial fission is required for dendritic spine plasticity and neuronal synapses [65,78,79,80,81,82].

#### 2.1.1. DRP1

Dynamin-related protein 1, also called dynamin-like protein, is a GTPase that translocates between the cytosol and the mitochondrial outer membrane, and it is regulated by phosphorylation and cytoskeleton interaction [83]. DRP1 promotes mitochondrial fission by forming a ring at mitochondria–ER contacts where membrane constriction occurs [6,14]. DRP1 also participates in peroxisome fission and mitochondrial calcium handling. The coordinated action of DRP1, peroxisomal membrane protein 11 (PEX11), and MFF promotes the constriction and division of peroxisomes [84,85]. In addition, DRP1 participates in the regulation of mitochondrial calcium levels. DRP1 knockout (KO) hippocampal neurons expressing a mutant amyloid precursor protein (APP) show mitochondrial calcium overload and increased APP-dependent neurotoxicity. These data suggest that DRP1 exerts a protective function in Alzheimer’s disease [86,87].

Although mitochondrial fission is associated with energy expenditure in brown adipocytes or excess nutrients and low ATP demand in β-cells [88], the increase in fission is usually associated with mitochondrial dysfunction or cancer invasion. For instance, ERK1/2-dependent DRP1 phosphorylation promoted by SIRT6 has been associated with increased mitochondrial fission and ovarian cancer invasion [89]. In colorectal, breast, and lung cancer and acute lymphoblastic leukemia, there is an increase in fission, and DRP1 phosphorylation is also associated with chemoresistance [90,91].

The increase in mitochondrial fission has been related to neurodegenerative diseases such as Parkinson’s disease (PD) and Alzheimer’s disease (AD). Regarding AD, the levels of DRP1 are frequently increased in postmortem tissues while mitochondrial fusion proteins Mitofusin 1 (MFN1) and Mitofusin 2 (MFN2) are reduced in these tissues. One might also conclude that AD may be associated with decreased mitochondrial fusion [92].

DRP1-associated dysfunctions are related to autophagy deficiency [93] or an increase in Radical Oxygen Species (ROS) levels [94]. The untreated diabetic condition, mimicked by prolonged exposure to high glucose levels, causes mitochondrial fission and an increase in ROS production [94]. Recently, a very interesting study showed that spontaneous fission can occur in two mitochondrial sites: peripheral or midzone. The occurrence of peripheral fission is less frequent in a healthy mitochondrion and is associated with the loss of mitochondrial membrane potential and high ROS and calcium levels. The product of peripheral fission is directed to mitophagy, while daughter mitochondria from midzone fission undergo mitochondrial dynamics [19].

#### 2.1.2. MFF

Mitochondrial Fission Factor (MFF) was described as an accessory receptor for mitochondrial fission, and it has an additional role in peroxisome fission [18]. The analysis of several human tissues demonstrates that MFF is highly expressed in the heart, liver, brain, muscle, and stomach [18]. MFF is the predominant DRP1 receptor in mammals, and its deficiency is associated with developmental delay, peripheral neuropathy, and optic atrophy. MFF-associated mitochondrial fission occurs independently of FIS1. It was demonstrated that mitochondrial fission is compromised by DRP1 and MFF knockdown, but not by FIS1 deficiency [95].

Liu and Chan (2015) characterized the interaction between DRP1 and MFF and concluded that MFF interacts with only complex DRP1 forms (tetramer or higher order) and that these forms are necessary for efficient fission [96]. On the other hand, MiD49 and 51, other DRP1 receptors, can interact with monomeric forms of DRP1 [96].

#### 2.1.3. FIS1

Mitochondrial fission protein 1 (FIS1) was first identified as essential for mitochondrial fission in yeast. Based on that observation, for many years it was considered a receptor/adaptor of DRP1 in humans [97]. However, growing evidence shows that the role of FIS1 can be cell-type dependent and that it is not essential for DRP1-mediated fission [95,98]. The depletion of FIS1 in some cells leads to mitochondrial elongation [99], while in others it does not alter the morphology of these organelles [95].

Experiments with yeast deficient in fission machinery demonstrate that reconstitution with MFF/DRP1 restores fission capacity, whereas FIS1/DRP1 does not have this effect [100].

Studies by Yu and colleagues (2019) raised the possibility that FIS1-induced fragmentation is a result of fusion inhibition rather than fission activation [98]. In DRP1 KO HEK293 cells, FIS1 overexpression causes mitochondria fragmentation through MFN1, MFN2, and OPA1 interaction and inhibition of their GTPase activity [98]. Studies focusing on identifying new DRP1 inhibitors indicate that residues 60–66 of FIS1 show high homology to residues 49–55 of DRP1 and that the use of a peptide inhibitor for these regions decreases mitochondrial fragmentation and oxidative stress and inhibits apoptosis [101].

Recently, structural studies showed that the arm in the N-terminus (residues 1–9) of FIS1 is decisive for the IN and OUT conformation of this protein and is necessary for its interaction with DRP1. The absence of these residues in the arm increases the interaction with fusion-related proteins. These observations thus suggest that, depending on cellular conditions, FIS1 inhibits mitochondrial fusion by interacting with fusion proteins through its arm [102].

Also, the discrepant results regarding the effects of DRP1 and FIS1 can be explained by Kleele’s results (2021), which revealed several aspects of fission [19]. Assays of cellular localization showed that MFF forms bright foci at the constriction site of mitochondrial midzone fission while FIS1 is distributed throughout the OMM in non-dividing mitochondria. In mitochondria undergoing peripheral fission, FIS1 is enriched and, in the case of FIS1 depletion, peripheral fission rates are decreased [19]. Additionally, the role of FIS1 in mitochondrial peripheral fission can be protective, since cardiomyocytes lacking FIS1 undergo major apoptosis [19].

FIS1 depletion associated with mitochondrial elongation promotes mitochondrial DNA (mtDNA) damage and a senescence-associated phenotype. These observations also may be associated with the selective FIS1 function in mitophagy [103,104]. High expression of FIS1 has been reported in patient samples of oral melanoma, and the mitochondrial fission induced by FIS1 in lung cancer stem cells leads to autophagy and cancer cell survival [105].

#### 2.1.4. MiD49 and MiD51

Mitochondrial elongation factors 1 and 2 (MIEF 1/2) or mitochondrial dynamics proteins of 49 kDa (MiD49) and 51 kDa (MiD51) were found in a screening to identify new uncharacterized proteins [106]. MiD49/51 is a strong DRP1 receptor that does not localize at peroxisomes but is present at mitochondria–ER contact sites [107,108]. The first implication of the involvement of MiD49/51 in fission machinery was provided by observations made by Palmer and colleagues. In this regard, MiD49/51 overexpression for short time periods was found to cause mitochondrial fission. This finding was reinforced by other studies that show that double knockdown also leads to mitochondrial elongation [109,110]. Moreover, MiD49/51 promotes fission independently of FIS1 or MFF [110]. However, other studies point to a fission inhibition role of MiD49/51. Zhao and co-workers described MiD51 as a fusion promoter because elevated levels of this protein cause mitochondrial elongation, while its depletion promotes mitochondrial fragmentation [111]. These authors proposed that MiD51 interacts with DRP1 and sequesters it, thus inhibiting fission [111]. Furthermore, the same group characterized MiD49 and observed similar effects causing mitochondrial fusion [112]. Palmer and colleagues attributed the association of MiD49/51 overexpression and mitochondrial fusion to extended overexpression [107].

Experiments with DRP1 mutants revealed that DRP1 oligomerization is required for interaction with MFF but not MiD49/51, thereby suggesting that the latter recruits an immature or inactive form of DRP1, thereby decreasing the availability of active DRP1 oligomers to interact with MFF [96]. Moreover, Lóson and colleagues showed that DRP1 phosphorylation at S637 is necessary for complex formation with MiD49/51 [110].

It is interesting that although MiD49/51 does not participate directly in peroxisome fission, by binding and sequestering DRP1, it also inhibits peroxisome fragmentation [107]. This result indicates that MiD49/51 is a stronger DRP1 recruiter than MFF or FIS1 [107].

In this case, MiD49/51 could facilitate mitochondrial fusion because of its self-association that approximates two adjacent mitochondria, facilitating MFN1/2-mediated tethering of two mitochondrial outer membranes [107]. This scenario is reasonable considering that fusion and fission machinery are in the same mitochondria–ER contact sites [2]. Indeed, mitochondrial elongation caused by MiD49/51 overexpression requires the action of fusion mediators MFN1 and MFN2 [107].

Atkins and colleagues have proposed that the discrepancy in connection with the functional role of MiD49/51 proteins in mitochondria dynamics (whether MiD49/51 mediate fusion or fission) can be attributed to cell type and the levels of MiD49/51 expression [113].

They also speculated that MiD49/51 can be used as a therapeutic target in diseases involving the dysregulation of mitochondrial dynamics, such as hyperproliferative conditions like cancer and pulmonary arterial hypertension (PAH) [113]. In PAH, for example, fission exceeds fusion, and the levels of MiDs are elevated, and, consequently, there is protection from apoptosis [114]. The silencing of MiD49/51 promotes mitochondrial elongation and a decrease in proliferation [114]. On the other hand, the inhibition of MiDs in acute ischemia–reperfusion injury decreases fission and protects the heart against cell death [115].

Studies of tissues from patients with ovarian cancer show that high expression of MiD51 is associated with poor prognosis. In contrast, in pancreatic cancer, MiD49 is considered a tumor suppressor [116,117].

### 2.2. Mitochondrial Fission Protein Phosphorylation

#### 2.2.1. DRP1 Phosphorylation

Two main phosphorylation sites have been described for human DRP1, namely S637 and S616. Several kinases are related to the phosphorylated forms of DRP1 S616. CDK1/cyclin B1 is the main kinase described, and this phosphorylation is very important because it occurs in the mitotic phase and is required for the equal distribution of mitochondria for daughter cells [118]. CDK5 also phosphorylates S616, but the consequence of this modification is still controversial. While some authors observed fission inhibition in post-mitotic neurons upon CDK5 phosphorylation at a DRP1 S616 equivalent residue in rats [119], the results of other studies indicate that phosphorylation by CDK5 at the equivalent serine residue in mice induces mitochondrial fission, which is associated with neuronal death upon NMDA-induced injury [120].

In a model of high glucose stimulation, mitochondrial fission is increased and associated with DRP1 S616 phosphorylation by ERK1/2 in vitro [121]. In human pancreatic cancer, DRP1 S616 phosphorylation by ERK2 and mitochondrial fragmentation is related to tumor growth [122]. This phosphorylation is associated with a series of negative consequences. For instance, Yuan and colleagues (2021) showed that hypoxia leads to ERK1 activation, DRP1 S616 phosphorylation, mitochondria fragmentation, and amyloid-β (Aβ) production [123].

Also, MAPK1 phosphorylated DRP1 at S616 and induced mitochondrial fragmentation and dysfunction in striatal cells of a Huntington’s disease model [124]. Studies with rat models of hypertension-induced encephalopathy showed that PKCδ phosphorylates DRP1 at S579 (equivalent to human S616) promoted mitochondrial fragmentation and neuronal cell death [125].

Finally, PTEN-induced kinase 1 (PINK1) also phosphorylated DRP1 at S616, which is necessary for autophagy independent of Parkin and is relevant for Parkinson’s disease (PD) [126].

Phosphorylation at T595 has been described in a model of PD. In this case, a mutated leucine-rich repeat kinase 2 (LRKK2) protein, which is frequently found in PD, phosphorylated DRP1 at T595, increased GTPase activity and mitochondrial fission, and led to exacerbated autophagy [127].

S637 phosphorylation is commonly associated with an inhibitory post-translational modification of DRP1 because this residue is located at the DRP1 GTPase effector domain (GED), and its GTPase activity would be inhibited after phosphorylation. However, the effect of S637 phosphorylation is rather controversial because some studies showed DRP1 inhibition [128] while others related S637 phosphorylation to fission activation [90,129].

This residue can be phosphorylated by protein kinase A (PKA) and B (AKT), leading to fission inhibition [130,131]. This notion was reinforced by studies using mitochondria depolarizing agents, which promoted an increase in calcium levels and activation of the phosphatase calcineurin. Calcineurin thus dephosphorylates DRP1 S637 and promotes DRP1 translocation to mitochondria, allowing fission to take place [132]. On the other hand, the dephosphorylation of S637 by PGAM5 inhibits fission [133]. Recently, Valera and colleagues (2021) demonstrated crosstalk among the DRP1 phosphorylation sites in mice. They showed that the phosphorylation of murine S600 (equivalent to S637 in humans) occurs before that of S579 (equivalent to S616 in humans) and that it is enough to induce an increase in p-S579 levels. In mice, they attribute the discrepancy in previous results to the effect of S600 phosphorylation on DRP1 isoforms and also to the requirement of the downstream phosphorylation of S579. In the absence of S579 phosphorylation, mitochondrial elongation can be observed after S600 phosphorylation [134].

Phosphorylation at S637 also occurs after A-kinase anchoring protein (AKAP)1 activation in rats with streptozotocin (STZ)-induced diabetes. This activation is accompanied by mitochondrial fission, podocyte damage with loss of mitochondrial membrane potential, a decrease in ATP synthesis, and increased ROS levels, and thus podocyte apoptosis [135]. A similar response to hyperglycemic stress has been reported in podocytes and endothelial cells, but this time, the phosphorylation of DRP1 at S600 (equivalent to S637) and mitochondrial fission were mediated by Rho-associated coiled-coil containing protein kinase 1 (ROCK1) [136].

In primary mouse chondrocytes, TANK-binding kinase 1 (TBK1) phosphorylated DRP1 at S637, thus attenuating TNF-α-induced apoptosis [137]. These authors suggested that this phosphorylation by TBK1 is necessary for autophagy. TBK1 also phosphorylated S412 at the DRP1 middle domain (MD) and S684 in the GED upon innate RNA sensing. These phosphorylations also inhibit DRP1 activity and are important for antiviral immunity [138].

#### 2.2.2. MFF Phosphorylation

MFF was identified in a screening for AMPK substrates. MFF contains two possible sites for AMPK phosphorylation. Although S172 was found to be a direct substrate of AMPK, the phosphorylation signal of MFF is lost only when S155 is concomitantly mutated with S172. AMPK is activated in response to mitochondrial stress, and it triggers mitophagy. Therefore, MFF phosphorylation can induce mitochondrial fragmentation to allow mitophagy to take place. This process occurs, for example, when there is an electron transport chain dysfunction, with a decrease in ATP levels. AMPK is activated, and it phosphorylates MFF, culminating in mitochondrial fragmentation [139].

As occurs for DRP1, MFF is also specifically phosphorylated in mitosis. The protein kinase PKD phosphorylates S155, S172, and S275 induce mitochondrial fission in mitosis but not during interphase [140].

#### 2.2.3. FIS1 Phosphorylation

**Figure 2:**
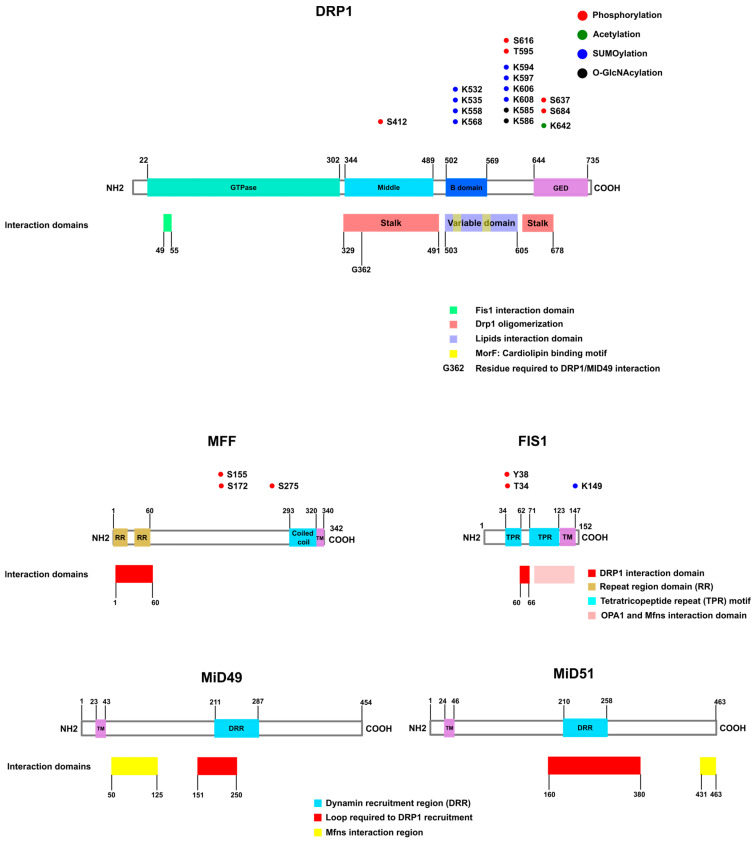
Mitochondrial fission proteins. The main players in mitochondrial fission are DRP1 and its mitochondrial adaptors. DRP1 is a dynamin-like GTPase that comprises the dynamin type G domain at the N-terminus (green), a middle domain (light blue), a B domain (blue), and a GTPase effector domain (GED) (magenta) at the C-terminus. Several post-translational modifications occur at DRP1, such as phosphorylation (represented in red), acetylation (green), SUMOylation (blue), and O-GlcNAcylation (black). The interaction regions in DRP1, where DRP1 interacts with its adaptors or itself, described to date are shown. FIS1 interacts with the dynamin-type G domain on DRP1. DRP1 oligomerization occurs via intermolecular stalk interaction. The stalk of DRP1 comprises the middle domain and the GED. The variable domain (also called insert B) contains the lipid interaction residues and the cardiolipin interaction region. A glycine residue in the stalk domain is required for DRP1 interaction with MiD49. MFF presents two domain repeats (R1 and R2), a coiled-coil domain (CC), and the transmembrane segment (TM). Interaction with DRP1 occurs via RR1 and RR2. FIS1 is formed by two tetratricopeptide repeat (TPR) motifs and a transmembrane domain. FIS1 interacts with DRP1 via residues located at the TPR motif. Also, the region in FIS1 that interacts with Mitofusins and OPA1 is shown. The regions of interaction with DRP1 are presented in FIS1 and MFF. MiD49/51 contains a transmembrane domain at the N-terminus and a Dynamin Recruitment Region (DRR). The region of interaction between MiD49 or MiD51 and Mitofusins is also shown [95,96,98,101,143,144,145,146,147].

FIS1 Y38 phosphorylation by Met tyrosine kinase, a receptor for hepatocyte growth factor (HGF), promotes mitochondrial fission and facilitates hepatocellular carcinoma (HCC) tumor growth [141]. This modification increases DRP1/FIS1 interaction. In addition, phosphorylation at Y34 occurs, and it also increases the affinity for DRP1 and induces mitochondrial fragmentation. This phosphorylation is mediated by DNA-PKcs in acute kidney injury and oxidative stress in renal endothelial cells after ischemia/reperfusion injury [142] (Figure 2).

### 2.3. Mitochondrial Fusion Proteins

Mitochondrial fusion is regulated mainly by GTPase dynamin-related proteins MFN1 and MFN2 located at the OMM, and autosomal dominant optic atrophy 1 (OPA1) at the inner mitochondrial membrane (IMM). Mitochondrial fusion requires both MFN1 and OPA1, while MFN2 is not essential for this function [148].

Mitofusins are mitochondrial outer transmembrane GTPases that participate in mitochondrial fusion and in the maintenance of contacts between membranes. The large N-terminus domains of Mitofusins are located at the cytosol, and they contain the GTPase domain, which is followed by heptad repeat (HR) 1; two transmembrane domains; and then a C-terminus, coiled-coil HR2, which, according to classical models, is also exposed towards the cytosol [15,149].

Interestingly, bioinformatic analysis and biochemical assays have found only one transmembrane domain, so the HR2 domain would be exposed to the intermembrane space (IMS). Moreover, it has been proposed that HR2–HR2 domain interaction in the IMS would permit Mitofusin oligomerization, and redox-sensitive cysteine Cys 684 and Cys 700 are key to promoting mitochondrial fusion [150].

Mice lacking MFN1 or MFN2 are embryonically lethal. This observation points to these molecules playing a key role in embryonic development [151].

Human Mitofusins are 62% identical and 77% similar to one another, and their expression levels vary depending on the tissue. Moreover, several cells express both proteins, thereby indicating that they do not have the same biological function. Both MFN1 and MFN2 depletion lead to mitochondrial fragmentation in mouse embryonic fibroblast (MEF) cells, although in a different way and intensity [151]. While MFN1 shows higher GTPase activity and a greater effect on mitochondrial fusion than MFN2, both proteins can form homo- or heterodimers to mediate fusion [151].

After GTP binding, a rearrangement of residues around the nucleotide-binding pocket occurs, losing intramolecular HR1–HR2 interactions and exposing the HR2 to the cytosol. This allows the dimerization between both HR2 domains from two opposite Mitofusins. After dimerization and GTP hydrolysis, a conformational change induces the close state, dragging the opposite membranes close, allowing fusion [152,153,154,155,156].

**Figure 3:**
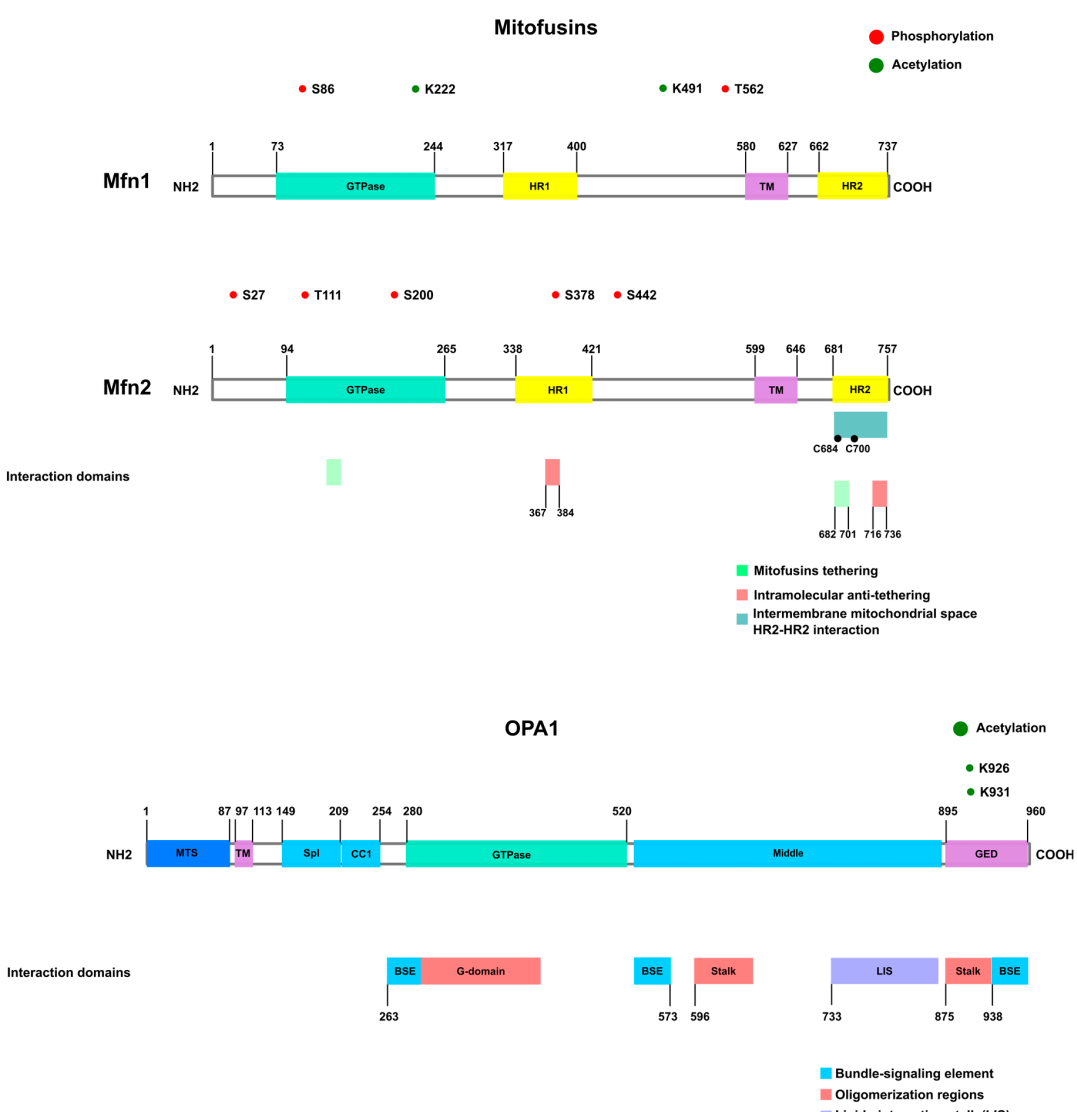
Mitochondrial fusion proteins. Mitofusins are dynamin-like GTPases comprising the GTPase domain at the N-terminus, followed by HR1, the transmembrane region (TM), and HR2 at the C-terminus. The phosphorylated and acetylated residues are shown in red and green, respectively. The HR domains are essential for the anti-tethering and pro-tethering states. Intramolecular interaction between HR1 and HR2 is associated with the anti-tethering conformation (red). Upon nucleotide binding, a conformational change exposes the HR2 domain to the cytosol, allowing interaction with the HR2 from adjacent mitochondria and tethering (represented in green). The GTPase domain also participates in the interaction for fusion [152,153,154,155,156]. An alternative model proposed that HR2–HR2 domain interaction occurs in the intermembrane mitochondrial space [150]. Isoform 1 of OPA (the long form) presents the transmembrane region at the N-terminus, followed by a coiled coil (CC), G domain (GTPase), middle domain, and GED. OPA1 oligomerization involves the G domain, middle domain, and GED domain [152,156,157].

This proposed model does not fit with Mattie and colleagues’ conclusions, whereby trans-dimerization occurs in the oxidative environment of the IMS that allows the formation of hydrogen bridges between two HR2s from two adjacent Mitofusins [150] (Figure 3).

Several lines of evidence point to overlapping functional roles for MFN1 and MFN2, as well as exclusive functions. We will explore the specific functions of each protein below.

#### 2.3.1. Mitofusin 1

MFN1 plays a key role in OMM fusion that is related to its GTPase activity. Surprisingly, in contrast to OPA1 and MFN2, no human pathologies related to MFN1 mutations have been identified to date. This observation thus suggests that MFN1 plays an important role during gestation and that the loss-of-function is incompatible with human life [158].

The metabolic effect of MFN1 KO or knockdown depends on the cell type. For example, the fragmentation observed in some tissues, such as the liver and myocardium, from MFN1 KO-diabetic mice is associated with an increase in mitochondrial respiration related to lipid metabolism. In the liver, MFN1 KO can have a protective role against insulin resistance because lipids are used as the main energy source. These mice also show an increase in Complex I activity [47]. However, in skeletal muscle or neurons, the reduction or ablation of MFN1 expression leads to a decrease in the mitochondrial oxidative phosphorylation system (OXPHOS), while in brown adipose tissue (BAT), MFN1 KO has no effect on energy expenditure (for review, see [159]).

The shift from oxidative to glycolytic metabolism and mitochondrial fission has been described in several types of cancer (lung, ovarian, colorectal, and pancreatic, among others). However, fusion and oxidative metabolism were also found in pancreatic cancer, suggesting that changes in mitochondrial dynamics favor or support cancer development (for review, see [159]). In hepatocellular carcinoma (HCC), MFN1 downregulation is related to metastasis and poor prognosis. MFN1 depletion triggers the epithelial–mesenchymal transition, and the inhibition of MFN1 GTPase activity via FUNDC2 interaction is associated with metabolic reprogramming and tumor growth [160,161]. In addition to causing metabolic changes, MFN1 can affect cell death. In this regard, MFN1 KO MEF cells are reported to be resistant to apoptotic stimuli [160]. This resistance was attributed to the inefficient accumulation of BAX on the OMM with incorrect curvature caused by hyper-fragmentation.

Both MFN1 and MFN2 interact with BAX and BAK. However, only the former is related to apoptosis. After apoptotic stimuli, BAK dissociates from MFN2 and associates with MFN1, facilitating BAK oligomerization and then cytochrome c release and cell death [162,163,164].

#### 2.3.2. Mitofusin 2

While MFN1 is the main player in OMM fusion, MFN2 plays a more prominent role in metabolism regulation. MFN2 stimulates mitochondrial respiration, glucose oxidation, and mitochondrial membrane potential, and its effects are not related to GTPase or fusion activity. MFN2 appears to regulate the expression of subunits of all OXPHOS complexes. In this regard, MFN2 loss-of-function reduces the subunits from OXPHOS Complexes I, II, III, and V, and the overexpression of this Mitofusin increases the expression of subunits related to Complexes I, IV, and V [165].

MFN2 expression was induced in mouse muscle during myogenesis, and its depletion led to mitochondrial fragmentation, reduced glucose oxidation and cell respiration, loss of mitochondrial membrane potential, and proton leak [3].

MFN2 is also present at the ER, and it interacts with MFN1 or MFN2 located on the OMM [166]. MFN2 location at the ER is essential for MFN2 function related to mitochondrial metabolism. This interaction is important to maintain contacts between these two organelles [166,167,168] and for Ca^2+^ and PS transfer from the ER to mitochondria, thus increasing mitochondrial metabolism and phosphatidylethanolamine (PE) synthesis [73,169].

Furthermore, MFN2 also plays a key role in maintaining mitochondrial contacts with other membranous structures, such as LDs and melanosomes (for review, see [170]). Although the effect of MFN2 at these contacts is still being studied, MFN2 knockdown frequently reduces the contacts between mitochondria and other membranes.

MFN2, but not MFN1, plays a crucial role in the ER stress response. MFN2 loss of function in MEF cells leads to an increase in ER chaperone levels. Also, ER stress caused by thapsigargin or tunicamycin induces an increase in MFN2 expression [171]. The depletion of MFN2 in MEF cells causes basal activation of the PERK pathway, and, despite exacerbation of the unfolding protein response (UPR), these cells are not able to activate apoptosis and/or autophagy [172].

Mutations in MFN2 cause the autosomal dominant neuropathy named Charcot–Marie–Tooth type 2A (CMT2), characterized by chronic axonal neuropathy [173,174]. So far, more than 100 different MFN2 mutations have been described in CMT2 patients [173,174,175,176,177]; however, the molecular defects are not completely understood.

MFN2 mutations have already been associated with reduced axonal mitochondrial activity [178,179], but mitochondrial morphology changes are not so clear and are dependent on the specific mutation and the cell type studied. Electron micrographs of the sural nerve from biopsies of two patients showed small, electron-dense, and swollen mitochondrial axons [176]. On other hand, cells from patients with different MFN2 mutations did not show any mitochondrial changes in morphology [180], with the exception of fibroblasts derived from patients with R364W mutations that showed changes in mitochondria–ER contacts. R364W cells present an increase in the length of mitochondria–ER contacts and also an increase in the distance between organelles. The cholesterol metabolism and lipid droplet formation also increased in these cells [180]. Studies with MFN1 and MFN2 double KO cells showed that the overexpression of MFN2 mutants induces several different phenotypes confirming that endogenous MFN1 or non-mutated MFN2 can compensate for the defects induced by MFN2 mutations [181]. Moreover, a study with a small MFN2 agonist showed effects only when endogenous MFN1 was present [179]. As CMT2A patients are heterozygous, endogenous MFN1 and non-mutated MFN2 can compensate for defects in mitochondria morphology and respiration.

Most mutations detected in CMT2A are missense located at the GTPase or coiled-coil domains (Figure 3). R94 was found mutated in several studies (R94Q or R94W), as were R280H and R104W or R364W (between GTPase and HR1) and T105M [177].

Rocha and colleagues have proposed that MFN2 activity is mediated by the interaction between Methionine 376 and Histidine 380 with Aspartate 725 and Leucine 727 and the phosphorylation at Serine 378 [179]. They showed that a MFN2 agonist mimics the peptide–peptide interface of MFN2, allosterically activating it, and promoting mitochondrial fusion. The agonist does not show activity above the mutants but increases the activity of functional MFN2 rescuing the defects associated to CMT2A [179].

Mitochondrial dysfunction is associated with insulin resistance, and muscles from obese or type 2 diabetic patients show a reduced expression of MFN2 [182].

MFN1 and MFN2 act together to maintain glucose homeostasis by preventing TFAM loss and consequently preserving the mtDNA content in β-cells. Neither MFN1 nor MFN2 is individually essential to preserve β-cell function, but together they coordinately promote the regulation of glucose levels and glucose-stimulated insulin secretion [183].

In BAT from mice on a high-fat diet, the deletion of MFN2, but not MFN1, reversed mitochondrial dysfunction, leading to an increase in insulin sensitivity and resistance to obesity [184]. In contrast, this deletion in skeletal muscle and liver promotes mitochondrial fragmentation, a decrease in oxidative phosphorylation, glucose intolerance, and enhanced hepatic gluconeogenesis [185,186]. In mouse skeletal muscle, MFN2 protected mitochondria from damage. The reduction of MFN2 in that tissue led to a decrease in autophagy and caused the accumulation of dysfunctional mitochondria, a phenotype related to aging [187].

MFN2 has a protective role in liver diseases such as non-alcoholic steatohepatitis (NASH). Liver-specific ablation of MFN2 in mice results in inflammation, fibrosis, and liver cancer. These effects are related to the role of MFN2 in mitochondria–ER contacts and are associated with a reduction of PS transfer and thus decreased phospholipid synthesis, and consequently ER stress [73].

MFN2 KO in neurons leads to a reduction in neuritic growth, and this is reversed by the expression of an ER-targeted MFN2 [169]. MFN2 is involved in mitochondrial quality control by participating in Parkin recruitment and then autophagy [188].

#### 2.3.3. OPA1

Autosomal dominant optic atrophy (OPA1) is the mammalian ortholog of yeast Mgm1p, and it is associated with mitochondrial fusion [189]. The name refers to the observation that mutations in this gene cause inherited optic neuropathy [190,191].

OPA1 is essential for mitochondrial fusion. However, independent of this function, it also has effects on cristae formation and mtDNA maintenance. Indeed, in the case of energetic stress, cells harboring OPA1 fusion-deficient mutants are able to sustain ATP levels and retain cristae structure [17,192,193].

Delettre and colleagues reported eight mRNA isoforms for OPA1. All OPA1 variants contain a cleavage site, S1, for mitochondrial processing peptidase, and some of them have an additional cleavage site, S2 [194].

After translation, OPA1 splicing variants are processed and can produce several proteins (at least five distinct bands in SDS-PAGE from 80–100 kDa are identified). OPA1 is translated with a matrix-targeting signal (which is cleaved by the mitochondrial processing peptidase (MPP) in the mitochondrial matrix) followed by the transmembrane domain (Figure 3). After MPP cleavage, the mature OPA1 isoform (also named L-isoform) in the matrix can be further processed, giving rise to short variants. This processing can occur through cleavage sites S1 (Exon 5) and S2 (Exon 5b) [195,196].

Song and colleagues conducted assays using MEF OPA1 null cells expressing individual OPA1 splicing variants. They observed that cells expressing OPA1 isoforms 3, 5, 6, or 8 showed predominantly fragmented mitochondria, and none of them expressed the long OPA1 protein form (L-OPA1) [196].

The expression of the long form of OPA1(L-OPA1) is always accompanied by a short form (S-OPA1). In complementation assays, both short and long forms were found to be necessary for efficient mitochondrial fusion, thereby indicating that S1 cleavage is essential for OPA1 activity related to fusion. Indeed, assays using proteoliposome (OPA1 + phosphatidylcholine) and liposome (cardiolipin and phosphatidylcholine) to simulate the IMM and OMM showed heterotypic tethering between cardiolipin and L-OPA1 before GTP hydrolysis and fusion. This tethering can be facilitated by S-OPA1. Also, this assay revealed that cristae maintenance is due to trans-OPA1 tethering when low levels of cardiolipin are present [197].

The origin of short isoforms was initially associated with mitochondrial membrane potential loss and mitochondrial fragmentation [195]. However, it is now known that, in addition to inducing S1 cleavage, CCCP, a mitochondrial membrane potential uncoupler, causes the degradation of L-OPA1 forms. Moreover, CCCP does not induce S2 cleavage [196]. Despite morphological differences, the cristae structure, amount of mtDNA, and energy efficiency can be regulated for any OPA1 isoform, regardless of length, independently of protein products [198].

The regulation of OPA1 is highly complex, and the generation of small isoforms depends on the activity of at least three proteases, namely m-AAA; a matrix metalloprotease named OMA1 (overlapping with m-AAA protease); the inner membrane rhomboid PARL (presenilin-associated rhomboid-like) protease; and the inner membrane protease, named i-AAA protease YME1L (ATP-dependent protease yeast mtDNA escape 1-like). YME1L cleaves OPA1 at the S2 site while OMA1 uses S1 [195,196,199].

OPA1 cleavage by OMA1 is increased under different types of cellular/mitochondrial stress and is regulated by autocatalytic proteolysis, allowing the reversibility of fusion inhibition [200]. The ablation of OMA1 in mice leads to hepatic steatosis, decreased energy expenditure, and impaired thermogenic regulation associated with transcriptional changes in genes involved in lipid and glucose metabolism [199].

YME1L, on the other hand, cleaves OPA1 under increased OXPHOS activity, and it is degraded in ATP-depleted cells. YME1L deficiency is associated with heart failure, impaired eye development, and spinal cord degeneration. OMA1 and YMEL1 can be reciprocally degraded depending on mitochondrial conditions (ATP levels, membrane potential state, OXPHOS activity, etc.) [46,201,202,203,204].

PARL is related to the antiapoptotic function of OPA1, such that PARL ablation in mice leads to muscle loss, progressive cachexia, and death at only 8 weeks of age, and these effects are associated with extensive lymphocyte apoptosis. Indeed, PARL cleaves OPA1, thereby generating soluble short OPA1 forms that are located in the IMS. OPA1 plays an anti-apoptotic role because it maintains the cristae structure by preventing cytochrome c release via the presence at IMS of the oligomers formed after PARL cleavage [192,205]. PARL also has a key role in the respiratory chain because it regulates coenzyme Q biosynthesis and Complex III activity, and its deficiency causes alterations in mitochondrial structure, neuronal necrosis, and a phenotype similar to that of Leigh syndrome [206].

Since OPA1 was first linked to dominant optic atrophy, a disease that affects retinal ganglion cells and the optic nerve, several other diseases related to the nervous system, namely spastic paraplegia, multiple sclerosis-like syndrome, parkinsonism, and dementia, have been associated with OPA1 gene mutations (for review, see [207]).

Mutations in the OPA1 gene are found in 32–90% of patients with autosomal dominant optic atrophy (ADOA) [208,209,210,211]. More than 150 mutations have already been identified [211], and most of them cause premature truncations of OPA1 and are located at the GTPase or GED domains [208,212]. V903G, R290Q, R904D, N158S, and E907G are among the most frequent mutations [190,211]. Also, there are mutations in splicing sites resulting in changes in OPA1 isoform expression. A change in a splicing site can lead to exon skipping or amino acid residue substitution (D438Y or D438H) that can affect protein dimerization [211].

Studies in patient-derived cells have documented aberrant cristae structure and mitochondrial fragmentation (G300E or R290Q mutations, for example). On the other hand, OPA1 58 delta (with a frame-shift deletion of 58 residues in the GED domain) showed normal filamentous mitochondria, although OPA1 protein expression was almost half compared to control cells [212]. Cartes-Saavedra and colleagues (2023) showed that GED is not necessary for OPA1 oligomer formation and fusion, but it is necessary for GTPase activity [213].

A mutation found in lymphoblastoid cell lines derived from Asian ADOA-affected patients led to P400A change; decrease in mtDNA; and defects in respiratory capacity, ATP synthesis, and mitochondrial membrane potential. These conditions caused increased oxidative damage and apoptosis [214].

OPA1 deficiency is also related to inflammation and myopathies. OPA1 ablation in the skeletal muscle of mice results in mitochondrial fragmentation, reduced respiration, ER stress, and the secretion of systemic inflammatory markers and FGF21 [215,216,217]. OPA1 KO mice also show improved glucose tolerance and are resistant to age- and diet-induced insulin resistance [216,217]. The OPA1 role in browning is not well understood because mice with selective KO of OPA1 in brown adipose tissue present increased levels of FGF21 and enhanced browning of white adipose tissue [218]. On the other hand, OPA1 overexpression has been associated with white adipose tissue expandability and the improvement of glucose tolerance and insulin sensitivity [219].

OPA1 is also important for cancer development, although its function or final effect is not clear. In breast cancer, OPA1 upregulation is associated with poor prognosis. In addition, in hepatocellular carcinoma (HCC) and lung adenocarcinoma, OPA1 expression determines the response to chemotherapeutics, and OPA1 knockdown sensitizes cells to the treatment. OPA1 involvement in tumor development may be related to its role in angiogenesis [220,221,222].

### 2.4. Mitochondrial Fusion Protein Phosphorylation

#### Mitofusin 1 and Mitofusin 2 Phosphorylation

The mechanisms that regulate Mitofusin activity are poorly understood. To date, only two MFN1 residues have been described as phosphorylated, affecting the activity or organization of the protein. MFN1 is phosphorylated at T562 by ERK1/2 after apoptotic stimuli, leading to MFN1 oligomerization inhibition and mitochondrial fragmentation [162].

MFN1 is also phosphorylated at S86 by PKC IIB, leading to a decrease in MFN1 GTPase activity and, consequently, the inhibition of fusion [223].

For MFN2, on the other hand, several target residues and kinases have been documented. However, these phosphorylations are associated with MFN2 degradation and not the regulation of MFN2 activity [188,224,225].

JNK1 and PINK1 phosphorylate MFN2 at residues S27 and S442, respectively. These phosphorylations permit MFN2 ubiquitination by Parkin and subsequently mitophagy activation [188].

MFN2 is also phosphorylated at S442 by AMPK upon energy stress, leading to mitochondrial fission, autophagy, and an increase in the number of contacts between the ER and mitochondria [226]. As mentioned before, AMPK also phosphorylates MFF. Hu and colleagues suggested that, during acute stress, AMPK phosphorylates MFN2, inducing autophagy and survival. However, under prolonged stress, the association of AMPK with MFF is increased, thereby reinforcing mitochondrial fission and autophagy [226].

Recently, MFN2 was identified as a substrate of mTORC [227]. MFN2 S200 is phosphorylated, and it leads to an increase in GTPase activity and respiration. This phosphorylation is particularly interesting in the context of cancer because cancer cells usually show dysregulated fusion/fission processes. The authors propose that MFN2 S200 phosphorylation in cancer tissues increases MFN2/Pyruvate kinase isoform 2 (PKM2) interaction, thereby favoring mitochondrial fusion and the switch between OXPHOS and glycolysis. This interaction would be a mechanism with which to protect mitochondria from over-fragmentation. Table 2.

## 3. Other Post-Translational Modifications of Mitochondrial Dynamics Proteins

In addition to phosphorylation, several other post-translational modifications have been reported in mitochondrial dynamics proteins. Additional DRP1 post-translational modifications include acetylation [229], o-GlcNAcylation [230,231], and SUMOylation [232,233]. FIS1 is SUMOylated [234] and acetylated [235]. MFN1 and MFN2 are SUMOylated [236], and MFN1 acetylation has also been reported [42,237]. OPA1 is acetylated [238] (Figure 2 and Figure 3).

### 3.1. Mitochondrial Dynamics Protein Acetylation

Protein acetylation is a reversible introduction of an acyl group at the ε-sidechain of a lysine residue in a target protein. This process neutralizes the lysine charge and favors alterations in protein conformation [239]. A wide family of acetyltransferases and deacetylases has been reported [240]. Surprisingly, total cellular proteome studies reveal that 20% of mitochondrial proteins are acetylated, among which 80% are mitochondrial metabolic enzymes [241]. Additional research evidences an inverse relation between proteome acetylation and mitochondrial metabolism [242]. Thus, acetylation–deacetylation post-translational modifications play a critical role in sustaining the cellular metabolism balance.

Overnutrition induced by a high-fat diet (HFD) results in an increase in fatty acid oxidation and the accumulation of acetylCoA. In addition, excessive oxidative stress can decrease the NAD+ pool required to activate deacetylases. Thus, under metabolic stress, protein acetylation is enhanced. Several studies suggest that increased acetylation of mitochondrial dynamics proteins promote mitochondrial fragmentation and decreased mitochondrial function.

In this regard, HFD-induced obesity increases DRP1 acetylation at K642 and promotes cardiac dysfunction in mice. Moreover, DRP1 K642R, a deacetylation mimic mutation, suppresses DRP1 oligomerization and mitochondrial fission in cardiomyocytes. These findings suggest that DRP1 acetylation impairs the function and dynamics of mitochondria through exacerbated fission in these organelles. In line with this, OPA1 acetylation and mitochondrial fragmentation have been reported in pathological heart hypertrophy models, including db/db mice, pressure overload transverse aortic constriction (TAC), and angiotensin chronic infusion [229].

SIRT3, a mitochondrial deacetylase, decreases OPA1 acetylation and promotes its GTPase activity, thereby activating mitochondrial fusion in the heart [238]. Additionally, SIRT3-dependent OPA1 deacetylation promotes the differentiation and OXPHOS activation of human cardiomyocytes derived from human induced pluripotent stem cells (hiPSC) [243].

MFN1 acetylation has been explored in MEF cells and skeletal muscle. Under nutrient starvation, MFN1 is associated with HDAC6 deacetylase, and it increases mitochondrial elongation. Moreover, HDAC6 ablation suppresses mitochondrial elongation in MEF cells and the *tibialis* anterior muscle of HDAC6 KO mice upon fasting. Also, a mutation in the potential acetylation lysine residue K222 promotes mitochondrial elongation [237]. Future studies should seek to validate MFN1 acetylation at K222 using mass spectrometry.

On the other hand, studies suggest that the acetylation of mitochondrial dynamics proteins serves as a label for mitochondrial quality control. Under acute mitochondrial stress induced by antimycin, rapid and transitory mitochondrial network elongation improves metabolism and decreases oxidative stress [11]. These effects on mitochondrial morphology and function are associated with increased acetylation of MFN1, which allows the activation of mitochondrial quality control [42]. MFN1 acetylation at K491 facilitates interaction with MARCH5, a ubiquitin ligase. Under acute mitochondrial stress, the mitochondrial network is hyperfused, and the quality control of the mitochondrial network is activated. MARCH5 ubiquitinates acetylated MFN1 and promotes its turnover under conditions of mitochondrial stress [42].

In line with these observations, a study using MEF cells and hiPSC demonstrated that acetylation facilitates FIS1 ubiquitination and degradation. Cellular reprogramming to pluripotency requires mitochondrial fission and acetyl carboxylase 1 (ACC1)-dependent *de novo* lipid synthesis. ACC1 expression decreases intracellular acetylCoA, whereby FIS1 is deacetylated and mitochondrial fission is activated. Hence, crosstalk between lipid metabolism and mitochondrial fission mediated by ACC1 allows human fibroblast reprograming [235] (Table 3).

These results give rise to the question: are there specific acetylation sites in mitochondrial dynamics proteins that cause mitochondrial fission and mitochondrial metabolism suppression, and are others associated with mitochondrial turnover?

### 3.2. SUMOylation of Mitochondrial Dynamics Proteins

SUMOylation is an enzymatic and reversible conjugation of small ubiquitin-like modifier (SUMO) proteins at lysine residues of target proteins. Mediated by SUMO 1 or SUMO 2/3, the reaction can be reversed by several isoforms of SUMO-specific peptidases (SENPs).

Recent work reports that FIS1 is SUMOylated at K149 by SUMO2/3, and this modification suppresses FIS1 localization at the OMM. Moreover, the authors demonstrated that FIS1 deSUMOylation at K149 by SENP3 allows mitophagy to take place in deferoxamine-treated cells [234]. Consistent with this finding, it has been reported that neuronal ischemic stress promotes PERK-dependent SENP3 lysosomal degradation. Under this condition, DRP1 remains SUMOylated, and the mitochondrial network is elongated. In contrast, reperfusion restores SENP3 levels, promotes DRP1 deSUMOylation and mitochondrial fragmentation, and triggers apoptosis [233].

Additional work has revealed a close relationship between DRP1 S616 phosphorylation and SUMOylation. DUSP6 is a redox-sensitive phosphatase that dephosphorylates DRP1 at S616 and promotes mitochondrial elongation. Under oxidative stress or ischemic/reperfusion, DUSP6 is deSUMOylated by SENP1, ubiquitinated, and degraded. Thus, DRP1 phosphorylation at S616 is sustained under stress conditions, and the mitochondrial network is fragmented [244]. This mechanism supports the notion that deSUMOylation by SENP3 protease might modulate mitochondrial architecture and metabolism.

In contrast, several studies describe that SUMOylation promotes mitochondrial fission and apoptosis. DRP1 is SUMOylated by SUMO1 during staurosporin-activated apoptosis. This modification helps stabilize mitochondria–ER contacts to promote the activation of apoptosis. Furthermore, DRP1 SUMOylation is required for mitochondrial calcium uptake and cristae remodeling [245].

In addition, DRP1 SUMOylation appears to play a key role in tumor growth and metastasis. Cancer progression has been associated with an increase in mitochondrial fission and a decrease in mitochondrial fusion protein expression. In breast cancer cells, SYNJ2BP-COX16 (a readthrough of synaptojanin 2 binding protein and COX16) SUMOylation regulates mitochondrial morphology through DRP1 SUMOylation and DRP1 phosphorylation at S616. Hence, mitochondrial network fragmentation occurs, thereby contributing to cancer cell proliferation and metastasis [246].

Furthermore, a recent report demonstrates that mitochondrial fusion proteins MFN1 and MFN2 are SUMOylated by SUMO 1 under metabolic stress induced by CCCP treatments. The authors propose that the SUMOylation of these Mitofusins helps to select and separate depolarized mitochondria from the mitochondrial network. Moreover, this step may be necessary to recruit mitophagy machinery at the mitochondrial membrane [236].

Emerging evidence points to an association between SUMOylation and acetylation post-translational modifications. In this regard, in fasting conditions, SENP1 deSUMOylase is translocated to mitochondria, and it removes SIRT3 SUMOylation at K288 and promotes SIRT3 deacetylase activity [247]. Moreover, a SIRT1 K223R (mouse homolog K288 residue) mutation that suppresses SIRT1 acetylation improves metabolism. Thus, SIRT1 K223R transgenic mice show less weight gain than control mice under HFD-induced obesity [247].

In this regard, a report describes a metabolic link between protein SUMOylation, acetylation, and modulation of the metabolic reprogramming required for the development of memory lymphocytes T (T_M_). T_M_ cells show reduced levels of acetylated proteome compared to effector lymphocytes T (T_E_). Moreover, AMPK activates the SENP1-SIRT3 axis in T_M_ cells. Thus, YME1L1 deacetylation by SIRT3 reduces its protease activity, increases L-OPA1 levels, and promotes mitochondrial fusion and OXPHOS activation in T_M_ cells [248] (Table 4).

### 3.3. O-GlcNAcylation of Mitochondrial Dynamics Proteins

Nutrient overload increases oxidative stress and mitochondrial dysfunction and impairs OXPHOS activity [56,250,251]. Several studies have linked o-GlcNAcylation to decreased lipid and glucose metabolism. Recent work revealed that conditional inducible overexpression of GLUT-4 in the heart of diabetic mice promotes an increase in o-GlcNAcylation, mitochondrial dysfunction, and diastolic dysfunction [252]. Knockout mice for O-GlcNAc transferase (OGT), an enzyme that transfers GlcNAc from UDP-GlcNAc to protein threonine or serine residues, with HFD-induced obesity show an increase in lipolysis in visceral fat [253]. Moreover, a decrease in perlipin1 (Plin1) o-GlcNAcylation by OGT promotes its phosphorylation and lipolysis [254].

DRP1 o-GlcNAcylation has been detected in the heart of streptozotocin-induced diabetes in mice [230]. o-GlcNAcylation of DRP1 at T585 and T586 in cardiomyocytes decreases DRP1 S637 phosphorylation and increases mitochondrial fragmentation in cardiomyocytes [230]. In addition, the ablation of O-GlcNAcase (OGA), a glycosidase that removes o-GlcNAcylation, increases mitochondrial fragmentation, decreases the NDUFB8 complex I subunit, and promotes DRP1 o-GlcNAcylation [231]. A study suggests that OPA1 is also O-GlcNAcylated upon high glucose treatments. This modification is associated with dysfunction of both mitochondrial fission and OXPHOS activity. Further investigation should be directed to demonstrate the direct O-GlcNAcylation of OPA1 protein [255].

Taken together, these studies suggest that O-GlcNAcylation of mitochondrial dynamics leads to mitochondrial dysfunction. Hence, this post-translational modification could be a potential target for drug design.

## 4. Participation of Lipids in Mitochondrial Morphology

### 4.1. Mitochondrial Phospholipids

In addition to the mitochondrial dynamics proteins mentioned above, a specific phospholipid composition of the OMM and IMM is required for the transient rupture of the membrane bilayer through fusion and fission processes. In this regard, a proper phospholipid composition is important not for only maintaining membrane fluidity and curvature but also for the correct membrane binding of protein machineries that allow mitochondrial biogenesis and function [256,257,258].

Mitochondria cannot produce phospholipids such as phosphatidylcholine (PC), phosphatidylinositol (PI), or phosphatidylserine (PS), which are derived from other cellular organelles. However, they can produce cardiolipin (CL), phosphatidylethanolamine (PE), and phosphatidylglycerol (PG) using the lipid precursors phosphatidic acid (PA) and PS, which are imported from the ER [72].

### 4.2. Mitochondrial Phospholipid Composition

The phospholipid compositions of the OMM and IMM differ significantly. Most CL is localized in the IMM and accounts for 15–20% of the phospholipids present in this membrane [259], while PC is the most abundant phospholipid in both membranes [260]. This phospholipid composition generates unique mitochondrial membrane properties.

Whereas PC, PI, and PS are cylindrical-shaped phospholipids that promote bilayer formation, PE, CL, and PA are conical-shaped, and they impose negative curvature stress on the membrane, allowing the formation of non-bilayer structures that facilitate fission and fusion events and cristae organization [261].

Mitochondrial membrane fusion and fission can be modulated by non-bilayer phospholipid concentrations. It has been reported that cardiomyocytes from Barth Syndrome patients, in whom CL level is decreased, show enlarged mitochondria and abnormal cristae morphology [262]. In this regard, CL is needed for IMM fusion, via its interaction with OPA1 [197], and for mitochondrial fission, through protein–lipid interaction with the B-insert domain of DRP1, which enhances its oligomerization and assembly (Figure 2) [144,263].

At the IMM, PE plays crucial roles in maintaining the tubular morphology and respiratory function of mitochondria. Studies demonstrate that when mitochondrial PE content is reduced by ~20%, mitochondrial oxygen consumption and ATP production decrease, consistent with defects in OXPHOS Complexes I and IV [264]. In addition, cells lacking phosphatidylserine decarboxylase (PSD), the enzyme that catalyzes the formation of PE from PS in mitochondria, present mitochondrial fragmentation and a swollen morphology [265,266].

On the other hand, PA, a minor component of the mitochondrial membranes, is a negative regulator of mitochondrial fission through its interaction with DRP1. Mitochondrial phospholipase D (MitoPLD), an enzyme that catalyzes the formation of PA from phospholipids such as PC or CL, mediates the binding of DRP1 to the PA head group at the OMM. The activity of DRP1 is thus inhibited, thereby impairing mitochondrial fission [267]. In this regard, recent studies have demonstrated that several proteins involved in maintaining the lipid composition of mitochondrial membranes modulate mitochondrial morphology (Table 5).

Mitochondrial dysfunction induced by lipid dysregulation, either by alteration of synthesis, turnover, or transport, has been associated with the genesis and progression of several diseases. Detailed information about diseases triggered by defective mitochondrial membrane phospholipids can be found in the recent review by Dong et al. [268]. This section will focus on mitochondrial lipid transfer proteins (LPTs) and scramblases involved in the maintenance of mitochondrial PL levels.

**Table 5:** Proteins that sustain mitochondrial membrane lipid composition.

Protein	Function	Localization	Protein Loss-of-Function	Ref.
			Mitochondrial Phenotype	
PSD	Conversion PS to PE	Mitochondria	Mitochondrial fragmentation	[264,265]
MitoPLD	Conversion PA to PC and CL	Mitochondria	Mitochondrial fragmentation	[269]
StarD7	Lipid transfer activity, PC distribution into mitochondria	Mitochondria	Mitochondrial fragmentation, mitochondrial cristae alterations	[270,271,272]
ORP5	Transfer PS from ER to mitochondria	MCSs	Mitochondrial cristae alterations	[273,274]
ORP8	Transfer PS from ER to mitochondria	MCSs	Mitochondrial cristae alterations	[273,274]
MFN2	Tethering	MCSs, mitochondria	Mitochondrial fragmentation	[166]
PTPIP51	PE to MCSs	MCSs, mitochondria	-	[275]
MIGA2	Tethering, transfer PS from ER to mitochondria	LD–mitochondria contact, MCSs	Mitochondrial fragmentation	[74,276]
PLIN1	Scaffold	LD–mitochondria contact	Mitochondrial cristae alterations	[277]
PTPMT1	Phosphatase	Mitochondria	Mitochondrial fragmentation	[278]
VPS13A	Tethering	MCSs	Mitochondrial fragmentation	[279]
VPS13C	Tethering	MCSs	Mitochondrial fragmentation	[280]
VPS13D	Tethering	MCSs	Mitochondrial fragmentation	[34]
VAP-A	Tethering	MCSs	Mitochondrial fragmentation	[281]
PRELID1	Lipid transfer activity, supply PA for CL biosynthetic	Mitochondria	Mitochondrial fragmentation	[282]
TRIAP1	Lipid transfer activity, supply PA to the CL biosynthetic pathway	Mitochondria	Mitochondrial fragmentation	[282]
TAFFAZIN	Acyl transferase	Mitochondria	Reduced cristae density	[283]
PLSCR3	CL transference between OMM and IMM	Mitochondria	Mitochondrial cristae alterations	[284]
ABHD16A	PS to Lyso-PS	Endoplasmic reticulum, MCSs	Mitochondrial fragmentation	[8]

### 4.3. Lipid Transfer Proteins

**Figure 4:**
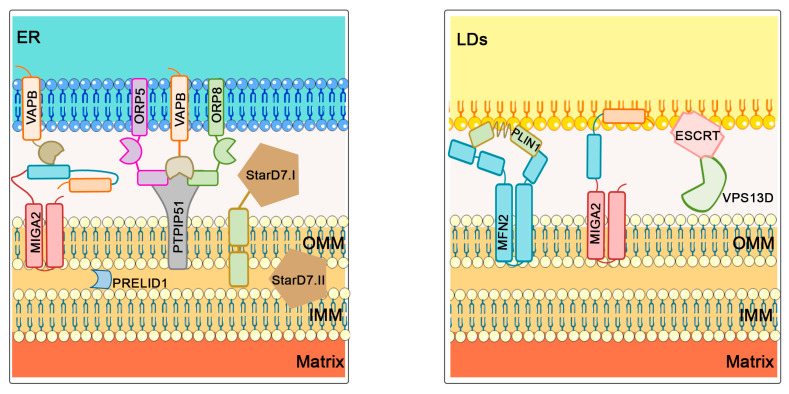
Phospholipid transport at ER–mitochondria and LD–mitochondria contact sites. MIGA2 binds mitochondria directly to LDs, and mitochondria to the ER by interacting with the ER protein VAP-A/B. ORP5 and ORP58 mediate the transfer of PS from the ER to mitochondria and interact with VAPB and PTPIP51 proteins, while StarD7 regulates PC distribution. Across the mitochondrial membranes, PRELID1 is responsible for PA transfer across the membranes, allowing CL biosynthesis in the IMM. In addition to MIGA2, the PLIN1 protein is also involved in the formation of LD–mitochondria contact sites by interacting with MFN2. VPS13D protein is known to interact with ESCRT on the surface of LDs, thereby also allowing FA transport.

LTPs play a major role in regulating the lipid composition of mitochondrial membranes by promoting non-vesicular lipid transport. The function of LTPs at mitochondrial contact sites has recently been addressed. LTPs contain a lipid-binding/transfer domain that include the Steroidogenic Acute Regulatory Protein-related Lipid Transfer (START); the oxysterol-binding protein (OSBP)-related proteins; or the proteins of relevant evolutionary and lymphoid interest (PRELI)-like domains, which act as transporters, tethers or lipid sensors at contact sites between mitochondrial membranes and the ER or LD membranes [285] (Figure 4).

The lipid transfer protein StarD7 regulates the distribution of PC within mitochondrial membranes and the ER. It is synthesized as a precursor form, StarD7-I, which contains a mitochondrial targeting sequence (MTS) and is cleaved by the mitochondrial peptidases MPP and PARL to produce the mature form StarD7-II [286]. StarD7 deficiency modifies mitochondrial network morphology and mitochondrial cristae structure, thereby causing mitochondrial dysfunction [270,271,272].

Two members of the OSBP family, the lipid transfer proteins ORP5 and ORP8, transfer PS from the ER to mitochondria and are required for sustaining mitochondrial morphology and respiration [273,274]. In addition, MFN2, which promotes mitochondria–ER tethering [287], also facilitates PS transfer from the ER to the mitochondria [73].

The protein tyrosine phosphatase-interacting protein 51 (PTPIP51) is reported to regulate CL levels by transferring its precursor PA via its TPR domain at mitochondrial–ER contact sites, and this transfer is independent of its interaction via its FFAT-like motif with the ER-VAMP associated protein (VAP) B [275]. Furthermore, PTPIP51 interacts with ORP5/8, thereby pointing to a functional link to lipid transfer [273].

MIGA2 is the only LD–mitochondria-binding protein that directly binds to both organelles via its amphipathic fragment. It is a transmembrane protein localized in LDs whose FFAT motif binds to the ER protein VAP-A/B, which promotes the formation of mitochondria–ER contacts and links reactions of de novo lipogenesis in mitochondria to triglyceride production in the ER [288]. MIGA2 also plays a role in mitochondrial morphology as its depletion leads to mitochondrial fragmentation [74,269]. Additionally, recent studies show evidence that MIGA2 binds and transports PS at mitochondria–ER contacts [74,276].

The perilipin 1 (PLIN1) protein is also involved in the formation of LD–mitochondria contact sites in BAT via its interaction with MFN2. In this model, depletion or knockout of MFN2 results in fewer LD–mitochondria membrane contact sites (MCS), altered lipid metabolism, and reduced FA oxidation by mitochondria [289].

Vacuolar protein sorting 13 (VPS13) A is involved in ER binding to mitochondria and the regulation of lipid transfer [279]. Loss-of-function mutations in VPS13A are associated with Chorea Acanthocytosis, a Huntington-like syndrome associated with abnormalities in red blood cells [290,291]. VPS13C depletion is linked to mitochondrial fragmentation, impaired respiration, and increased mitophagy, and it is associated with Parkinson’s disease [280]. An FFAT motif known to interact with VPS13A and VPS13C is present in both proteins [292], and it can be strengthened and reduced via phosphorylation of the FFAT motif [293,294].

VPS13D has a ubiquitin-binding domain required for mitochondria–ER contacts and mitochondrial clearance, and its loss results in enlarged mitochondria [295]. VPS13D is known to interact with ESCRT on the surface of LDs, transferring FA and contributing to membrane remodeling in that region [296]. Loss-of-function mutations in VPS13D cause autosomal recessive ataxia with abnormal mitochondrial morphology and reduced energy generation [297].

After being taken up into the mitochondria, phospholipids need to be distributed between the two mitochondrial membranes. The PRELID1/TRIAP1 protein complex transfers PA across the membranes, providing PA to the CL biosynthetic enzymes in the IMM [282].

### 4.4. Phospholipid Scramblases

The asymmetric distribution of phospholipids across the membrane bilayer necessary to preserve mitochondrial morphology depends on scramblases. These proteins facilitate lipid translocation from one membrane leaflet to another. Several scrambling proteins, such as PLSCRs, TMEM16 family members, and Xkr, facilitate the mixing of membrane lipids. In mitochondrial membranes, Phospholipid Scramblase 3 (PLSCR3) is responsible for CL transfer between the OMM and IMM [298,299]. It plays an important role in regulating mitochondrial morphology and respiratory function, as cells expressing a mutant PLS3 (F258V) have fewer and larger mitochondria with densely packed cristae as compared to control cells and have reduced oxygen consumption and intracellular ATP [284].

In addition to the transfer proteins mentioned above, mitochondrial morphology is regulated by the increased level of ER hydrolase ABHD16A, which mediates the hydrolysis of PS to lysophosphatidylserine (Lyso-PS) [300]. ABHD16A is required for ER-associated mitochondrial membrane constriction, and its depletion affects the recruitment of fission and fusion machinery, not only due to changes in Lyso-PS levels but also due to its ability to depalmitoylate mitochondrial transmembrane proteins [301]. Metabolic dysregulation of lyso-PS levels leads to the human genetic neurological disorder PHARC (polyneuropathy, hearing loss, ataxia, retinitis pigmentosa, and cataracts).

## 5. Pharmacological Approaches to Modulate Mitochondrial Architecture

Mitochondrial dynamics mediated by several fission and fusion events might be a strategic target with which to modify cellular metabolism. Finding new drugs to modulate mitochondrial architecture is an emergent field. In recent years, there have been reports of some compounds that modulate mitochondrial shape and metabolism.

Imeglimin is an antidiabetic drug structurally related to metformin that improves insulin sensitivity and mitochondrial function and decreases hepatic steatosis of high-fat, high-sucrose diet-treated mice [302,303,304]. This compound increases insulin release in obese db/db diabetic mice [305]. Moreover, this compound also modifies the mitochondrial morphology in pancreatic β-cells of db/db mice [305]. Studies in hepatic HepG2 cell lines show that like metformin, Imeglimin activates similar cellular signals, including AMPK activation and a decrease in mitochondrial respiration [306]. Interestingly, Imeglimin, as opposed to metformin, promotes the expression of Complex I and Complex III mitochondrial subunits [306]. Further investigations are required to elucidate the molecular mechanism of action of Imeglimin in mitochondria.

Mdiv-1 is a quinazoline-derived allosteric DRP1 inhibitor [307]. This compound promotes mitochondrial fusion, and it has been suggested that it protects from mitochondrial dysfunction caused by metabolic diseases and ischemic stroke [308,309,310,311]. Despite these reports, this compound shows Complex I off-target inhibition, and it impairs mitochondrial and cytosolic calcium homeostasis [312]. Also, several studies describe toxic cellular effects in some cells [313]. A recent report describes a novel DRP1 inhibitor, DRP1i27, that interacts with DRP1 and decreases its GTPase activity. This compound promotes mitochondrial elongation and protects from ischemic and reperfusion injury [314].

P110 is a seven-amino acid peptide that decreases the DRP1/FIS1 interaction and increases mitochondrial elongation [101,315]. In addition, this compound reduces oxidative stress and preserves mitochondrial respiration in LPS-treated cardiomyocytes [316]. In vivo studies demonstrated that p110 treatment for 10 or 24 days improves the motor and locomotion function of amyotrophic lateral sclerosis (ALS) mice. These mice carry the G39A SOD1 mutation that promotes oxidative stress, muscle atrophy, mitochondrial dysfunction, and cristae alterations. Overall these parameters were improved after P110 treatment [317].

Recent investigations, using a screening of molecules that can interact with the switch I-adjacent grove (SWAG) on DRP1 have led to the discovery of a novel DRP1 inhibitor, SC9. This molecule increases mitochondrial elongation under a cellular stress condition, such as LPS stimulus [315].

A recent study screened the mitochondrial morphology effect of 10,275 compounds using a high-content live-cell imaging approach [318]. Using this approach, PFK15 (6-phosphofructo-2-kinase (PFKFB3) inhibitor) was identified as a mitochondrial fission inhibitor. Based on the structure of this pharmacophore, a novel mitochondrial fission inhibitor, named MIDI, was synthesized. This compound increases mitochondrial elongation in cells treated with different stressors, such as hydrogen peroxide or mitochondrial function disruptors. Furthermore, MIDI promotes mitochondrial elongation of MFN1 KO cells. Mechanistically, this compound covalently interacts with cysteine C367 in the stalk domain on DRP1 [318].

Furthermore, antioxidant molecules can increase mitochondrial elongation. SS-31 (elamipretide) is a mitochondrial-target tetrapeptide with antioxidant capacity, and it improves mitochondrial elongation and mitochondrial fusion and promotes mitophagy [319,320]. SS-31 has been used in several models of cardiovascular disease. In addition, it is being tested in several clinical trials focused on heart failure, mitochondrial myopathy, renal disease, and aging [321,322,323,324].

The repurposing of FDA-approved drugs has opened new perspectives for drug indications. Leflunomide, a drug used for the treatment of rheumatoid arthritis, increases mitochondrial fusion by activating MFN1 and MFN2 expression [325]. Studies propose that a reversion of fragmented mitochondria in cancer might be a potential therapy. In this regard, leflunomide promotes mitochondrial elongation and increases the survival of pancreatic cancer patients [326].

A recent approach demonstrates that direct modulation of mitochondrial fusion using the TAT-367-384Gly fusogenic peptide creates a potential drug to treat mitochondrial disease. This peptide decreases HR1–HR2 interaction of MFN1 or MFN2 and results in the exposure of the MFN1/MFN2 HR2 domain to the cytoplasm. The open conformation of the HR2 domain might interact with another HR2 domain in other mitochondria and promote mitochondrial fusion. This peptide reverses the mitochondrial defect observed in CMT2A pathology [156]. Moreover, pharmacophore studies focused on HR1–HR2 MFN2 domain interaction and HR1 S378 phosphorylation revealed that a small chimeric molecule (Chimera B-A/l) promotes MFN2-dependent mitochondrial fusion [179]. Based on this molecule, MiM111 was developed. This compound improves mitochondrial transport and promotes axonal regeneration in models of CMT2A pathology [327,328,329].

A recent report describes a small molecule MASM7, which promotes MFN1 and/or MFN2 tethering through HR2 domain interaction. MASM7 increases mitochondrial fusion and enhances mitochondrial respiration, mitochondrial potential, and ATP production [330]. On the other hand, another molecule, MFI8, suppresses Mitofusin tethering, promotes mitochondrial fragmentation, and decreases mitochondrial function [330]. Remarkably, the design of these molecules has been based on the classical Mitofusin topology. Studies that describe Mitofusins as single-spanning membrane proteins may be also used to design novel pharmacological approximations, through the cysteine (Cys 684 and Cys 700) targeting of Mitofusins or the HR2–HR2 interaction region localized in IMS [150]. These studies may open new chemical approaches to modulate mitochondrial architecture. Direct activation or inhibition of the MFN1/2 tethering mechanism emerges as a promising strategy to directly modulate mitochondrial function. Table 6.

## Figures and Tables

**Table 1:** Cellular components that modulate mitochondrial architecture.

Organelle	Functional Effect	Ref.
Endoplasmic reticulum	Mitochondrial dynamics, mitochondrial fusion and fission, endoplasmic reticulum marks fusion and fission sites, calcium transfer, autophagy, iron homeostasis, lipid transport	[2,6,20,23,72,73]
Lipid droplets	Lipid droplet expansion, fatty acid oxidation	[74]
Peroxisomes	Coordinate fatty acid metabolism, ROS scavenging	[33]
Endosomes	Endosome maturation, acidification of early endosomes	[36]
	Iron transfer	[75]
	Mitochondrial mRNA translation in axon	[38]
Lysosomes	Mitochondrial calcium transfer, lipid transport	[40]
Actin cytoskeleton	Mitochondrial dynamic, mitochondrial shape, mitochondrial positioning	[1,7]
Intermediate filaments	Mitochondrial shape	[7]
Tubulin	Mitochondrial transport, mitochondrial shape, mitochondrial positioning	[5,7,76]

**Table 2:** Phosphorylation sites of mitochondrial dynamics proteins.

Protein	Amino Acid Residue (Human)	Kinase	Mitochondria Phenotype	Ref.
DRP1	S616	ERK1/2	Fission	[121,122,123,228]
		CaMKI alpha	Fission	[129]
		ROCK1	Fission	[136]
		Cdk1/cyclin B	Fission	[118]
		CDK5	Fusion? *	[119]
			Fission	[120]
		MAPK	Fission	[124]
		PKC	Fission	[125]
		PINK	Fission	[126]
	S637	AKAP1	Fission	[135]
		ROCK1	Fission	[136]
		PKA	Fusion	[130]
		AKT/PKB	Fusion	[131]
		TBK1	Fusion	[137]
	S412	TBK1	Fusion	[138]
	S684	TBK1	Fusion	[138]
	T595	LRKK2	Fission	[127]
MFF	S155	AMPK, PKD	Fission	[139,140]
	S172	AMPK, PKD	Fission	[139,140]
	S275	PKD	Fission	[140]
FIS1	Y38	MET	Fission	[141]
	T34	DNA PKcs	Fission	[142]
MFN1	S86	βII PKC	Fission	[223]
	T562	ERK1	Fission	[162]
MFN2	S27	JNK	no changes	[224]
	T111	PINK1	Fission	[188]
	S200	mTOR	Fusion	[227]
	S378	PINK1	Fission	[179]
	S442	PINK1	Fission	[188]
		AMPK	Fission	[226]

* The authors could not confirm the direct phosphorylation and that the effect of fission inhibition is associated with a monomeric form of DRP1 accumulated in the cytosol.

**Table 3:** Acetylation of mitochondrial dynamics proteins.

Protein	Amino Acid Residue	Deacetylase	Effect on Mitochondria	Ref.
DRP1	K642	-	Fission	[229]
FIS1	-	-	Acetylation promotes FIS1 ubiquitination	[235]
MFN1	K491	-	K491R mutant promotes mitochondrial elongation and decreases MFN1 ubiquitination	[42]
	K222	HDAC6	Deacetylation promotes mitochondrial elongation	[237]
OPA1	K926/K931	SIRT3	Deacetylation promotes mitochondrial elongation	[238]

**Table 4:** SUMOylation of mitochondrial dynamics proteins.

Protein	Amino Acid Residue	SUMO	Effect on Mitochondria	Ref.
DRP1	K532/K535/K558/K568 /K594/K597/K606/K608 isoform 1	SUMO1, SUMO2/3	-	[232]
	K557R, K560R, K569R, and K571R Isoform 3		Fission	[249]
FIS1	K149	SUMO 2/3	Decreases FIS1 mitochondrial localization	[234]
MFN1/MFN2	-	SUMO2/3	Mitochondrial perinuclear aggregation	[236]

**Table 6:** Agents that target mitochondria and mitochondrial diseases.

Drug	Effect on Mitochondria Function	Disease	Ex Vivo and In Vivo Study Models		Clinical Trial	Ref.
			Dose	Study Models		
Imeglimin	Modulates OXPHOS, modulates mitochondrial morphology, antioxidant	Diabetes mellitus type 2	0.1; 0.25, 1; 3; 10 mM	HepG2 cells Mouse primary hepatocytes	EudraCT N. 2006-000909-29; 2011-004086-32; 2010-018580-42; 2010-023915-33	[305,306,331,332,333]
			250 mg/Kg 150 mg/Kg BID 200 mg/Kg BID	C57BLKS/J Iar-+Leprdb/+Leprdb mice (db/db); pancreatic islets KK-Ay/TaJcl (KK-Ay) mice, BKS; Cg-m +/+ Lepr db/Jcl (db/m) mice, BKS; Cg- + Lepr db/ + Lepr db/Jcl (db/db) mice		
SS-31 (elamipretide)	Improves mitochondrial respiratory capacity, increases mitochondrial supercomplex organization, stabilizes cardiolipin, modulates mitochondrial morphology, antioxidant, promotes mitophagy	Barth syndrome, dilated cardiomyopathy with ataxia syndrome (DCMA), heart failure, age-related macular degeneration, LHON, skeletal-muscle mitochondrial dysfunction in the elderly, diabetes	10 nM 100 nM 10 μM 25–125 μM	Human fibroblast strains from patients with biochemically and/or genetically confirmed DCMA, HK-2 (Human kidney epithelial cells), ARPE-19 (retinal pigment epithelia cell line), INS1 β-cells (rat insulinoma cell line)	NCT03323749;NCT03891875;NCT02976038;NCT05168774;NCT02814097;NCT03098797;NCT02914665;NCT02848313;NCT03323749;NCT02693119;NCT02245620;NCT02788747	[319,320,334,335]
			0.25–60 mg/kg	Taz^KD^ mice CB6F1 mice		
Quercetin	Enhances mitochondrial membrane potential, ATP levels, mtDNA PARP-1 inhibition; upregulates Nrf2 expression; modulates mitochondrial morphology; antioxidant	Osteoarthritis, cardiac hypertrophy, traumatic brain injury, hepatotoxicity	100 mg/Kg 20 mg/Kg	Wistar rats, Sprague-Dawley rats, SHRs, Wistar-Kyoto rats, ICR mice	None	[336,337,338,339,340]
			10–200 μM 50 μM	CHO cells U937 cells THP-1 cells HL-60 cells NB4 cells		
Mdiv-1	Modulates ROS, inhibits DRP1, reduces cytosolic Ca^2+^ overload, modulates mitochondrial morphology	Stroke, myocardial infarction, neurodegenerative diseases	1, 3, 10, 30 μM 5 μM 25 μM 50 μM	C57BL6/J mice, HL-1 atrial cells, GH3 cells, Primary ventricular myocytes, Neuronal primary culture, Sprague-Dawley rats	None	[341,342,343,344]
DRP1i27	Inhibits DRP1, modulates mitochondrial morphology	Ischemia–reperfusion injury	5, 10, 50 μM	HL-1 cells, human foreskin fibroblasts, iPSCs, CERA007c6 cell line	None	[314,345]
P110	Inhibits DRP1, blocks DRP1/FIS1 interaction and prevents/reverses excessive mitochondrial fragmentation, modulates mitochondrial morphology	Neurodegenerative diseases, Friedreich ataxia	1 μM	SH-5YSY cells, primary skin fibroblasts	None	[101,346,347]
			0.5, 1.0, 1.5 mg/Kg	C57BL/6 mice		
Idebenone (Coenzyme Q10 Analog)	Modulates OXPHOS, upregulates Lin28A, inhibits p52Shc PPARα/γ agonist	Friedreich ataxia (FA), MELAS, LHON, DMD, multiple sclerosis, Parkinson’s disease, Alzheimer’s disease, Huntington’s disease	5, 15 or 45 mg/kg 150–2250 mg/day	Cell culture, animal, human	NCT03891875;NCT02976038;NCT05162768;NCT02814097;NCT03098797;NCT02914665;NCT02848313;NCT03323749;NCT04689360;NCT02693119;NCT02245620;NCT02788747;NCT02805790;NCT01755858;NCT02367014;NCT01572909	[331,348,349,350,351]
MiM111	Activates mitofusin	Charcot–Marie–Tooth Disease, cardiomyopathy	100 nM 1 μM	Dermal fibroblasts (MFN2 T105M, MFN2 H361Y, MFN2 R274W), dermal fibroblasts, control patients, dorsal root ganglion (DRG) neurons were isolated from 8-week-old MFN2 T105M flox-stop transgenic mice	None	[328,329]
			30 mg/kg 50 mg/kg	MFN2 T105M mice: C57BL/6 Gt(ROSA)26 Sortm1 (CAG-MFN2*T105M)Dple/J)		

* BID: twice a day; DCM: MELAS (Mitochondrial Encephalopathy, Lactic Acidosis, and Stroke-like episodes) syndrome; LHON: Leber Hereditary Optic Neuropathy; SHRs: spontaneously hypertensive rats; Taz^KD^: tafazzin knockdown mice; DMD: Duchenne muscular dystrophy.

## Data Availability

Not applicable.
